# Modeling Chickpea Productivity with Artificial Image Objects and Convolutional Neural Network

**DOI:** 10.3390/plants13172444

**Published:** 2024-09-01

**Authors:** Mikhail Bankin, Yaroslav Tyrykin, Maria Duk, Maria Samsonova, Konstantin Kozlov

**Affiliations:** Mathematical Biology and Bioinformatics Lab, PhysMech Institute, Peter the Great St. Petersburg Polytechnic University, 195251 St. Petersburg, Russia; mikle.p.bankin@gmail.com (M.B.); tyrykinyarik@mail.ru (Y.T.); duk@mail.ioffe.ru (M.D.); m.g.samsonova@gmail.com (M.S.)

**Keywords:** artificial image objects, climatic factors, genomic prediction, chickpea, GWAS, convolutional neural network

## Abstract

The chickpea plays a significant role in global agriculture and occupies an increasing share in the human diet. The main aim of the research was to develop a model for the prediction of two chickpea productivity traits in the available dataset. Genomic data for accessions were encoded in Artificial Image Objects, and a model for the thousand-seed weight (TSW) and number of seeds per plant (SNpP) prediction was constructed using a Convolutional Neural Network, dictionary learning and sparse coding for feature extraction, and extreme gradient boosting for regression. The model was capable of predicting both traits with an acceptable accuracy of 84–85%. The most important factors for model solution were identified using the dense regression attention maps method. The SNPs important for the SNpP and TSW traits were found in 34 and 49 genes, respectively. Genomic prediction with a constructed model can help breeding programs harness genotypic and phenotypic diversity to more effectively produce varieties with a desired phenotype.

## 1. Introduction

The relevance of research in the field of agricultural crops is determined by the need to increase their productivity and resistance to adverse environmental conditions. One of the important crops is the chickpea, which plays a significant role in global agriculture due to its nutritional value and ability to fix nitrogen, thereby increasing soil fertility.

Chickpeas, being an accessible and rich source of nutrients, occupy an increasing share in the human diet every year. The chickpea is the second most widely grown food legume that is cultivated in more than 50 countries around the world, especially in West Asia and the Indian subcontinent. The chickpea provides nutritional nitrogen and high-quality protein for ≈15% of the world’s population. Thus, over the past 12 years, the share of chickpea consumers in the United States has increased by 236%, reaching 4.5% of the entire population [1].

The main targets for breeding in chickpeas include Fusarium wilt [2]; increased resilience to drought, heat, and cold; increased seed nutrient density; reduced dependence on inputs; and resistance to biotic stress [3]. The application of omics technologies in breeding has proven effective in chickpeas. Joint analysis of the available phenotypic and genomic data identified candidate markers for many agronomic traits. Among the recent results, 27 marker–trait associations (MTAs) linked to yield-related traits and heat stress were identified in [4] using the BLINK model, and 11 SNPs associated with *Fusarium* wilt resistance, dispersed across the genome, were found in [5]; several marker–trait associations (MTAs) associated with the drought-linked traits were identified in [6]. Breeding strategies based on genomic prediction to enhance crop productivity have been proposed.

Genomic prediction (GP) aims to predict the phenotype of an organism given the data on single-nucleotide polymorphisms (SNPs) [7]. The plethora of methods for genomic prediction can be classified in two groups. Linear methods such as BLUP perform well for additive traits. They model the phenotype as a function of contributions from different factors, e.g., individual markers, weather parameters, field conditions, etc. Dimensionality reduction methods are often used as a pre-processing step for genomic prediction methods [8]. On the other hand, nonparametric machine learning methods, e.g., Support Vector Machines, Random Forest, and Gradient Boosting Machine, can model nonlinear traits, providing tremendous flexibility to adapt to complicated associations between data and output [9].

The accuracy of prediction is affected by the quality and pre-processing of the phenotypic data, as well as the platform used to obtain genomic information, the population mating design, the intrinsic genetic architecture of the trait, the genetic structure of the population, how the genotype-by-environment interaction is dealt with, and the prediction method [10].

Among machine learning methods, convolutional neural networks (CNNs) provide the best ability to identify latent patterns or features from data and are best suited for image analysis [11,12]. Artificial Image Objects (AIOs) constitute a new concept for the representation of genomic data that can be used to encode large amounts of genomics data by considering individual genetic variants as pixels [13]. The advantages of AIOs are convenient straightforward visualization, compactness, and ability to apply vast number of techniques developed for image analysis and classification [14], particularly for the CNN [15]. The need for diverse methods for data representation and processing is particularly important due to the great increase in the numbers of genomic data such as *Cicer* super-pangenome [16]. Consequently, AIOs could be used by CNNs on regression and classification tasks [17].

Application of the CNN to the processing of AIOs makes it possible to calculate and visualize the impacts of different factors to the final model solution. Recently, increasing attention has been drawn to the internal mechanisms of convolutional neural networks, as well as the reason why the network makes specific decisions [18]. Several techniques have been introduced that include perturbation- and backpropagation-based approaches [19], gradient-based algorithms [20], and class activation maps [18]. A saliency map represents the spatial support of a particular class in a given image [21].

Though the application of existing methods may seem to be straightforward, the development of new approaches will broaden the range of available opportunities for the prediction of important plant characteristics. Modern machine learning algorithms overcome some of the shortcomings of classical methods, especially in the ability to model complex dependencies between data and output, but present new challenges, such as the selection of adequate data representation and model architecture that are addressed in this work.

The aim of the present research was to develop a model for chickpea productivity traits in the available dataset and to extract the most important features that influence the model solution.

The main contributions of this work are the following:

- A methodology was proposed that combines AIOs and modern ML algorithms;
- A model based on AIOs and a CNN was developed for the prediction of chickpea productivity traits using SNPs;
- The impacts of SNPs on the model solution were evaluated.

The developed approach can help breeding programs harness genotypic and phenotypic diversity to more effectively produce varieties with a desired phenotype.

## 2. Related Work

Machine learning methods have been used to increase the statistical power of GWAS [22], to detect epistatic interactions, to improve the polygenic risk estimate obtained using GWAS procedures [23] and for post-processing the results of GWAS analysis [24]. Recently, improved Random Forest (RF) [25] methods have been proposed and applied to GWASs, such as the T-Tree method and the ts-RF method [26], which optimize tree node separation rules; Szymczak (2016) [27] redefined the method for calculating importance scores; in [28], a hybrid RF algorithm was proposed.

The work of [29] was said to be the first study to apply a saliency map for a GWAS, and the missing values were treated as a new genotype resulting in four binary values for each SNP in soybean. The saliency value of each genotype was calculated as the maximum absolute value of gradients among those four coding channels; the population median value was used as a measurement of the SNP contribution.

Various methods for the interpretation of CNN models have been proposed recently. Class activation maps provide visual explanation for a single input [20,30] but are architecture-sensitive. Gradient-weighted Class Activation Mapping (Grad-CAM) uses the gradients of any target concept flowing into the final convolutional layer to produce a coarse localization map highlighting the important regions in the image for predicting the concept [20]. Score-CAM, unlike previous class-activation-mapping-based approaches, gets rid of the dependence on gradients by obtaining the weight of each activation map through its forward passing score on the target class; the final result is obtained by a linear combination of weights and activation maps [18]. Grad-CAM++ [30], the modification of Grad-CAM [20], generalizes CAM to models without global pooling layers. LayerCAM [31] may generate reliable class activation maps from combinations of class activation maps from different layers of the CNN.

DeepFeature, proposed in [32,33], was designed to transform omics data into a form that is optimal for fitting a CNN model. The input vector is transformed into a matrix using t-SNE, kernel PCA, PHATE, or UMAP, and the smallest rectangle containing all the elements is found using the convex hull algorithm; rotation is performed to align the image, and Cartesian coordinates are converted to pixel indices.

## 3. Materials and Methods

### 3.1. The Overview

**Figure 1:**
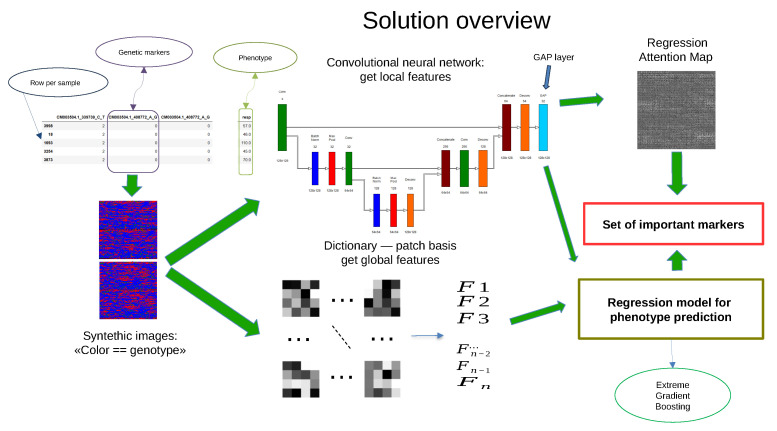
The overview of the research.

The methodology proposed in this work consists of several steps (see Figure 1):

1. Construction of artificial images for each accession by encoding information on the SNP values and climatic factors for limited period of time;
2. Building convolutional neural network for local feature extraction;
3. Dictionary learning and sparse coding for extraction of global features;
4. Construction of an extreme gradient boosting model for prediction of chickpea traits,
5. Evaluation of importance of input data for model prediction using the regression activation mapping technique.

These steps are described below.

### 3.2. Plant Material

A total of 407 chickpea *Cicer arietinum* samples collected in Ethiopia, Lebanon, Morocco, Turkey, India, Uzbekistan, and the Mediterranean region were phenotyped at the VIR Kuban experimental station in 2016. During the vegetative period, 36 phenological, morphological, agronomical, and biological descriptors were measured. Details on the phenotyping experiments, genotyping, and subsequent analysis resulted in 6642 SNPs that were presented in recent manuscripts [34].

**Figure 2:**
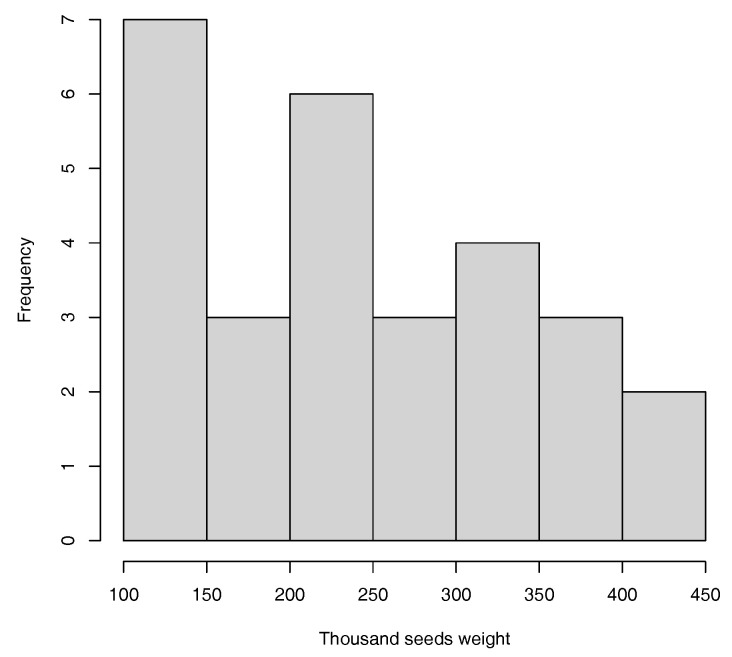
Histogram for TSW trait.

**Figure 3:**
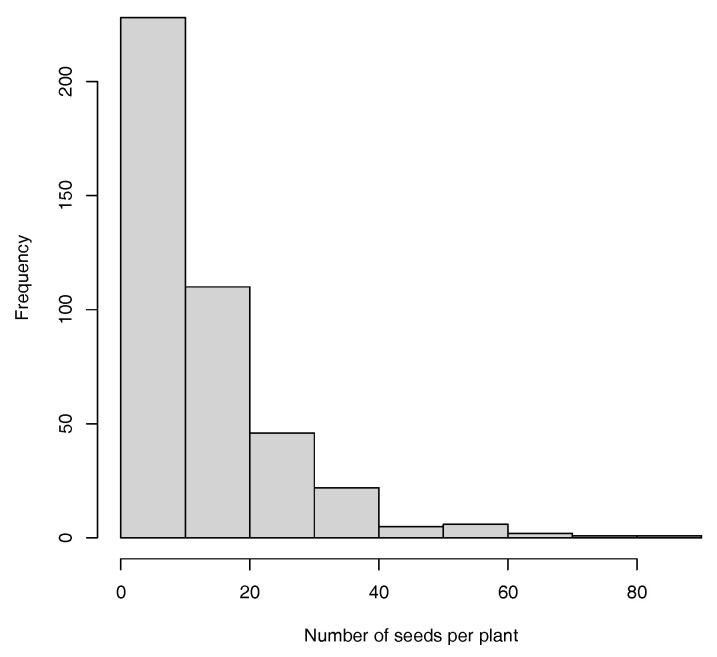
Histogram for SNpP trait.

In this work, two productivity traits were modeled, namely, the thousand-seed weight (TSW), which ranges from 110 to 440 g (see Figure 2), and the number of seeds per plant (SNpP), which ranges from 0 to 88 seeds (see Figure 3).

### 3.3. Artificial Image Objects

Artificial image objects were used to encode information on $V_{g}$ genomic features for each accession.

As the dimensions of AIOs may be selected arbitrarily, it was decided to set the number of rows and columns equal to 128 pixels. AIO I(x,y) can be represented as a matrix that is sequentially filled with the values of features from left to right in row-first manner; the list of features was reused from the start in order to fill the whole matrix. Each pixel value combines three channels, *R*, *G*, and *B*, for three pseudo-colors—red, green, and blue, respectively.

The value $f_{g}$ of SNP with index *k* was converted to a pixel value $i_{g}\left(x,y\right)$ according to (1):

(1) $$R=\left\{\begin{array}{cc} 1, & f_{g}=0\\ 0, & f_{g}\neq 0 \end{array}\right.\;G=\left\{\begin{array}{cc} 1, & f_{g}=1\\ 0, & f_{g}\neq 1 \end{array}\right.\;B=\left\{\begin{array}{cc} 1, & f_{g}=2\\ 0, & f_{g}\neq 2 \end{array}\right.$$

**Figure 4:**
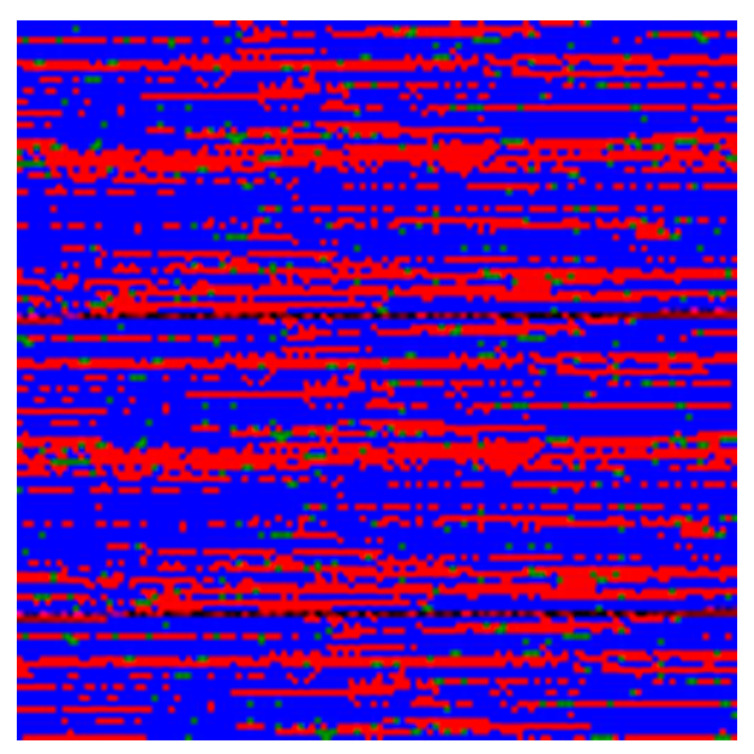
Example AIO. The size of the image is 128 × 128 px; here, the image is enlarged. Each colored square corresponds to one pixel. The color of each pixel is obtained by (1) for genomic factors.

The artificial image objects were constructed for all accessions. AIOs provide a convenient visualization for the data (see Figure 4). The colors of pixels in the AIOs are defined by Equations (1) for genomic factors.

### 3.4. Dictionary Learning and Sparse Coding

To account for the inter-relations between accessions in the dataset due to the genetic similarity that can impact the model performance, the additional features were extracted from the set of AIOs using the online dictionary learning and sparse coding approach [35].

The individual images are combined into one, which allows the algorithm to analyze all available features from all images and contributes to their closer integration. The combined image is then split into square blocks of the selected small dimension, for example, 16 by 16 pixels. Each image block can be represented as a weighted sum of the selected number, e.g., 16, of template images (called atoms) stored in a dictionary. In the training phase, template images are obtained from a set of blocks of real images, with the k-nearest neighbors method used to find similar templates and the singular value decomposition (SVD) method used to speed up the convergence of the iterative dictionary construction process.

The non-zero coefficients for each dictionary atom are distributed according the log-normal law, so the fitted means and standard deviations are used to calculate the vector of the microfeatures. The frequencies of atoms constitute the vector of the macrofeatures [36]. The concatenation of these two vectors was used as the numerical feature vector in the model.

### 3.5. Convolutional Neural Network

**Figure 5:**
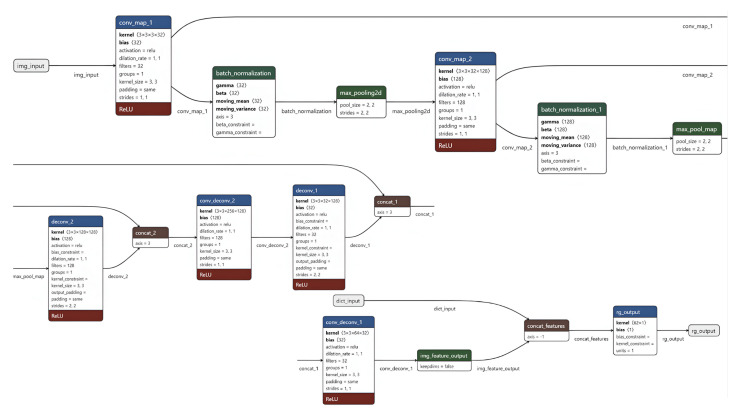
The architecture of CNN.

The model for phenotype prediction was built in the form of a convolutional neural network (see Figure 5) that has been improved in comparison to [17]. The CNN takes artificial image objects (img_input in Figure 5) and the numerical feature vector (dict_input in Figure 5) as the first and the second inputs, respectively. The size of the images was fixed at 128 by 128 pixels. Since the AIO contains three color channels, each filter is a collection of three kernels. Each kernel slides along the corresponding image channel; the results are combined into one feature map. The size of the filter kernel of each convolutional layer (Conv2D type) was found by adapting to the available experimental data. The values of the weights of the kernels were the learning parameters of the model. Each convolutional layer was followed by a subsampling layer (MaxPooling2D type), the purpose of which was to reduce the dimension of maps in order to enlarge features. Such filtering helps, among other things, to avoid overfitting. The formation of a new feature map is based on the Max Pooling operation, which is performed by selecting the maximum value from a subsample of a given size. The result is a set of 128 channels of 32-by-32-pixel feature maps. Next, using the operations of deconvolution, combining with intermediate feature maps to prevent loss of information, and using convolution, 32 channels of feature maps of the original size 128 by 128 pixels were obtained.

At the last stage, all feature maps were combined using a global average pooling layer (GAP) that transformed the maps into a feature vector, which was combined with the numerical feature vector. The combined vector was used to obtain a solution using a regression extreme gradient boosting model. The mean absolute error was used as a loss function. The neural network and regression model were trained simultaneously to obtain a consistent solution.

The network architecture includes 536,575 parameters and was implemented using the TensorFlow v2.12 and Keras v2.12 packages using a functional interface in the Python programming language.

### 3.6. Impacts of Different Factors to the Model Solution

Attention maps are a widely used method for explaining the results of convolutional neural networks. Classification and regression tasks use attention maps based on the values of network function gradients or the convolutional layers. In this work, we adapted the dense regression attention maps proposed in [37,38].

Attention maps were computed for input images obtained from genomic data of individual accessions and were of the same size. Thus, the pixel intensity characterizes the importance of a particular feature—SNP—for solving the model for a given sample. Consequently, in order to identify the most important features, it is necessary to determine those for which the average pixel intensity significantly differs from the averages for other features. We used Dunnett’s criterion [39], a well-known statistical test for many-to-one comparisons, to compare pixel averages.

## 4. Results

### 4.1. Dictionary Learning

The genetic similarities between accessions may affect the accuracy of the genomic prediction if not accounted in the model structure. The statistical parameters of the AIO decomposition coefficients according to the learned dictionary were used as the supplemental numerical features that characterize the population structure present in the dataset.

To optimize the solution, online dictionary learning method and the orthogonal matching pursuit method were adapted. The advantages of these algorithms are the reduced memory consumption during calculations and better accuracy. Several dictionaries were built for the dataset for error values of 0.005, 0.01, and 0.02 and for decomposition lengths of 10 and 20.

The dictionary with 16 atoms and an error value of 0.01 was selected, as it had the smallest number of missing coefficients.

### 4.2. Model for Number of Seeds per Plant

**Figure 6:**
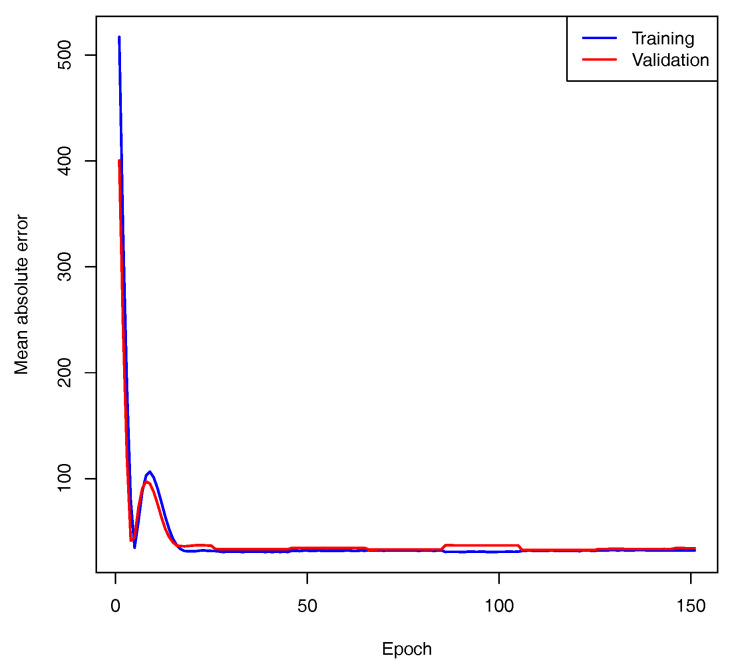
Convergence for SNpP trait.

**Figure 7:**
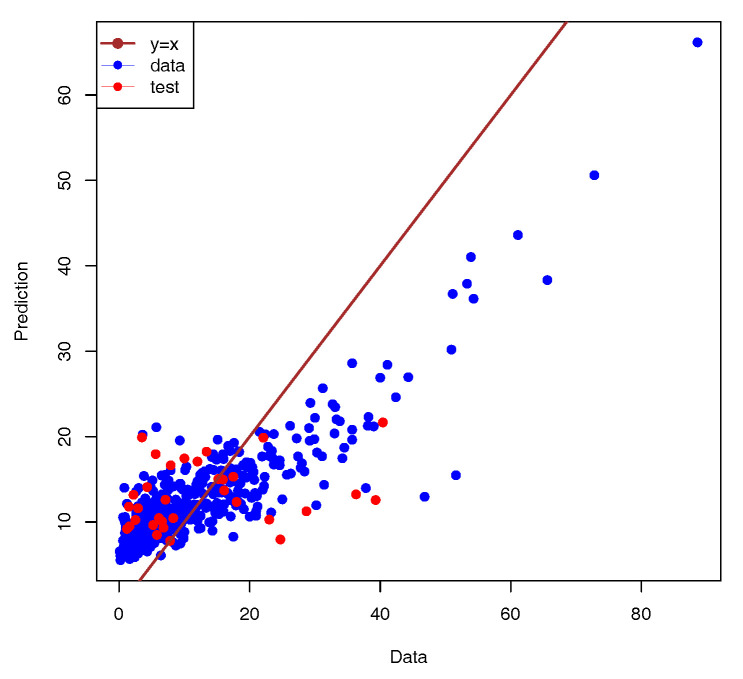
Comparison of measured and predicted number of seeds per plant. The data points used for training are marked with blue circles, and those from test set are drawn as red dots. The straight line represents the exact correspondence. The model accuracy was a=84%.

The model for the SNpP was trained using AIOs of 366 and 41 accessions for 10-fold cross-validation and control, respectively. The validation split parameter was set to 20%, and training was performed for 150 epochs using the mean absolute error as the loss function. The loss function curves for the training and validation subsets achieved approximately the same level, so the learning process was considered to be converged (see Figure 6). The model with the maximal accuracy for the validation set was considered to be the best and was selected for further investigation. The best model predicted the number of seeds per plant for the control dataset with a high accuracy of 84% (see Figure 7). This result is good for the task of predicting phenotypes using SNPs.

### 4.3. Important Features for Number of Seeds per Plant

**Figure 8:**
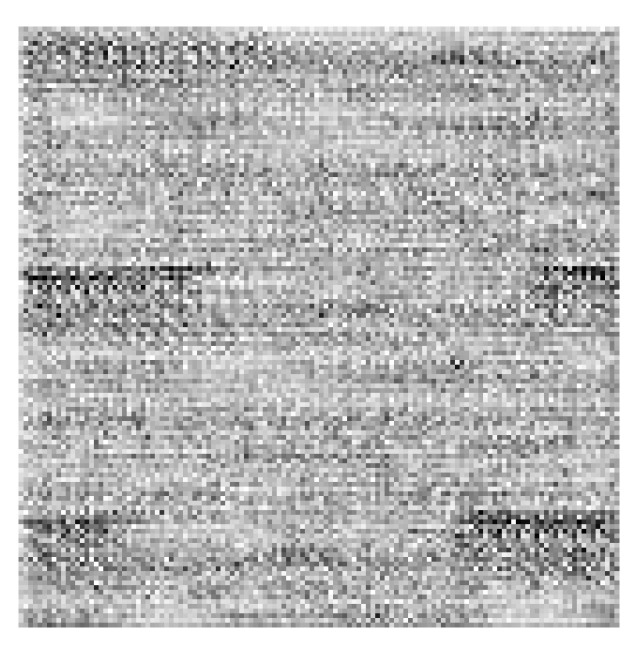
Example attention map for SNpP trait for individual accession. The intensity differences were increased for visualization purposes.

The attention maps were computed for each individual accession in order to identity SNPs with the largest impact on trait prediction. The individual map (see Figure 8) contains pixels of a high range of intensities, with several comparatively lighter areas containing potentially important SNPs.

A total of 99 SNPs were selected for the SNpP trait using the approach described in Section 3.6.

### 4.4. Functional Analysis of Identified SNPs for SNpP

For the SNpP, 34 out of 99 SNPs were located within known genes or within 1 kb flanking regions, and they most likely tag candidate casual genes (Supplementary Table S2). The functions of some of them are known.

The statistical significance of the difference in phenotype means was checked with the Student’s *t*-test between samples that were reference homozygous, alternative homozygous, and heterozygous for the identified SNPs. The difference in the SNpP was significant (*p*-value <0.05) for 23 out of 34 SNPs within genes and 1 kb flanking regions and for 28 more SNPs out of the remaining 65 identified for this phenotype (Supplementary Table S3).

The statistical significance of the difference in phenotype means was checked with a Student’s *t*-test between samples that were reference homozygous, alternative homozygous, and heterozygous for the identified SNPs. The difference for the TSW phenotype was significant (*p*-value <0.05) for 8 out of 49 SNPs within genes and 1 kb flanking regions and for 3 more SNPs among the rest of the identified 50 SNPs. The difference in the SNpP was significant (*p*-value < 0.05) for 23 out of 34 SNPs within genes and 1 kb flanking regions and for 28 more SNPs out of the rest of the 65 identified for this phenotype (Supplementary Table S3).

For the SNpP trait, an SNP (Chr1:7478974) was found within the *Ca_08000* gene encoding the biotin carboxyl carrier protein of acetyl-CoA carboxylase, which plays crucial roles in fatty acid metabolism [40].

An SNP (Chr1:7497743) was located 305 bp upstream of the *Ca_08003* genes for subunit 10 of the ER membrane protein complex, which inserts newly synthesized proteins into membranes [41] and performs esential functions, including protein and lipid synthesis [42]. An SNP (Chr1:26966675) was found downstream of the *Ca_20295* gene for the SEH1 protein. In *Arabidopsis thaliana*, SEH1 is implicated in immunity-related mRNA export [43]. An SNP (Chr1:19804162) was found within the *Ca_08855* gene encoding serine/threonine-protein kinase PCRK1-like required for plant immunity [44].

Three SNPs (Chr1:6354038; Chr1:6354059; and Chr1:6354082) were found within the *Ca_07897* gene for DEAD-box ATP-dependent RNA helicase 8. In *Arabidopsis thaliana*, this helicase modulates the formation of D-bodies, which are membraneless organelles that are made from liquid droplets of proteins and nucleic acids [45].

An SNP (Chr1:26891914) was located within the *Ca_20291* gene encoding zinc-finger homeodomain (ZF-HD) protein 2-like. All members of the subfamily of the ZF-HD homeobox genes are expressed predominantly or exclusively in floral tissue, indicating a likely regulatory role during floral development [46]. An SNP (Chr1:7592329) was found 213 bp downstream of the *Ca_08013* gene for AP2-like ethylene-responsive transcription factor AIL1 that is involved in the specification of meristematic or division-competent states [47].

An SNP (Chr1:31369599) was found within the *Ca_21855* gene controlling the long-distance transport of glucosinolates, which are defense compounds. The homologs of this gene in Arabidopsis control the loading of glucosinolates from the apoplasm into the phloem and into the seeds [48]. An SNP (Chr1:8130166) was found within the *Ca_08059* gene, which is the Glyma18g48580 homologue that encodes the subtilisin-like protease involved in plant immunity [49]. The *Ca_25478* gene, which contains an (Chr1:34314586) SNP, is a homologue of the *Arabidopsis thaliana* LEAF RUST 10 DISEASE-RESISTANCE (AtLRK10L1) gene. Mutation in this gene results in an abscisic acid (ABA)-insensitive phenotype in seed germination and seedling growth, as well as in reduced tolerance to drought stress [50].

An SNP (Chr1:27024341) was located within the *Ca_20299* gene for FAR1-RELATED SEQUENCE 5-like protein, which is a homologue in *Arabidopsis thaliana* that is essential for phytochrome A controlled far-red responses [51]. In addition to its essential role in light signaling, FAR1 also plays diverse regulatory roles in plant growth and development, including clock entrainment, seed dormancy and germination, senescence, chloroplast formation, branching, and flowering and meristem development [52].

An SNP (Chr1:6162693) was found within the *Ca_00701* gene for 1-aminocyclopropane-1-carboxylate oxidase, which catalyses the final step in ethylene biosynthesis—an important regulator of many developmental and physiological processes such as seed dormancy, germination, vegetative growth, flowering, climacteric fruit ripening, and senescence [53].

### 4.5. Model for Thousand-Seed Weight

**Figure 9:**
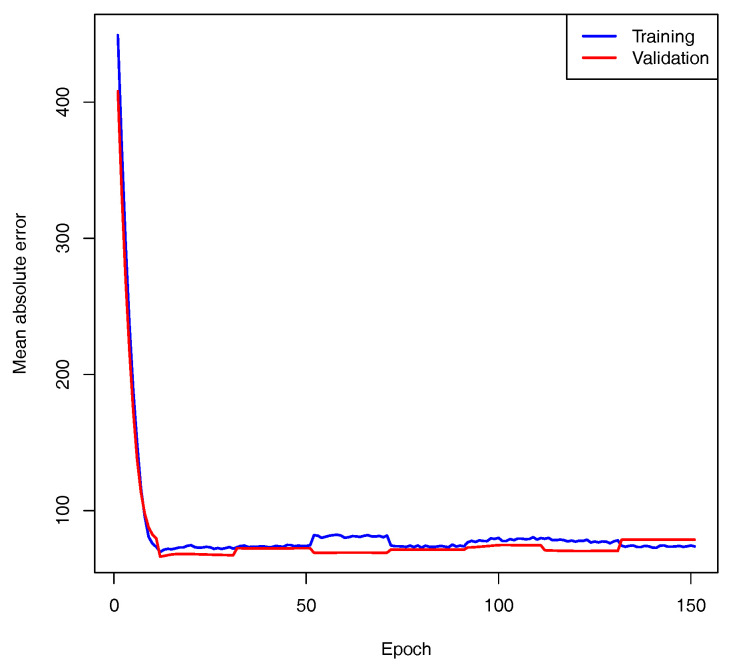
Convergence for TSW trait.

**Figure 10:**
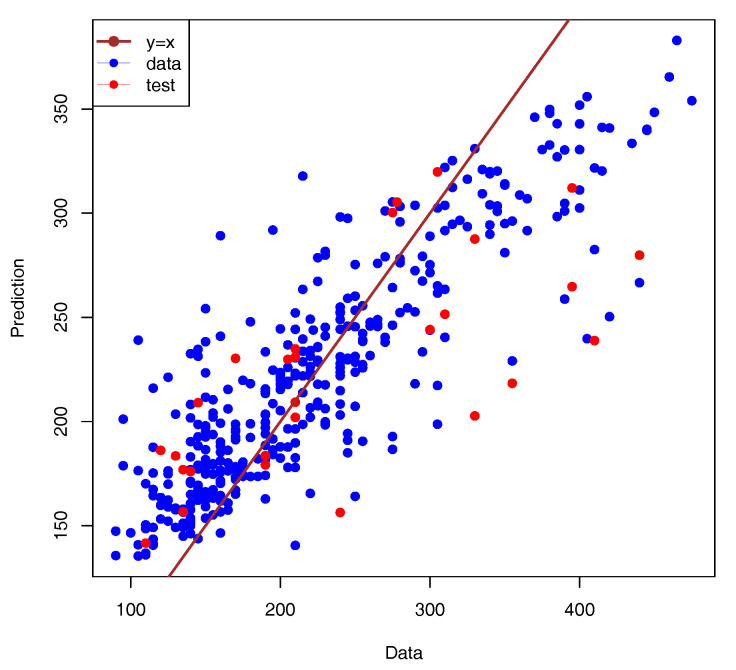
Comparison of measured and predicted thousand-seed weight. The data points used for training are marked with blue circles, and those from test set are drawn as red dots. The straight line represents the exact correspondence. The model accuracy was a=85%.

The model training for the TSW trait prediction was performed using 366 accessions for 10-fold cross-validation. The loss function and training parameters were the same as for the SNpP model, i.e, 150 epochs and a 20% validation subset. The convergence process is shown in (Figure 9). The best model was selected that had the maximal accuracy for the validation set. The best model predicted the TSW trait for the control dataset with a high accuracy of 85% (see Figure 10).

### 4.6. Important Features for Thousand-Seed Weight

**Figure 11:**
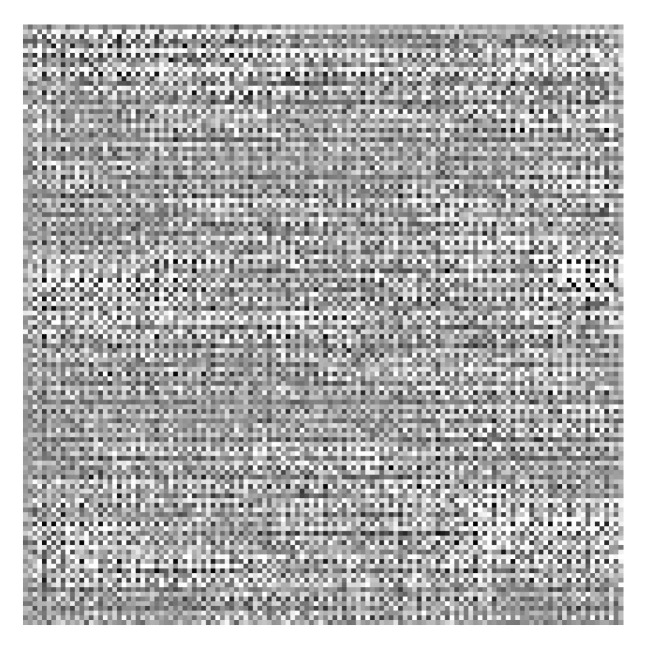
Example attention map for TSW trait for individual accession, the intensity was enhanced for visualization similar to Figure 8.

In order to find the most important SNPs, the attention maps (see Figure 11) were computed for each individual accession.

The individual attention map for this model (Figure 11) looked similar to that for the previous one (Figure 8). The gray levels of the pixels varied greatly, and several comparatively brighter islands can be identified that correspond to potentially important SNPs.

Following the same procedure described in Section 3.6, 99 SNPs were found for the TSW trait.

### 4.7. Functional Analysis of Identified SNPs for TSW

For the TSW trait, 49 out of 99 SNPs were located within known genes or within 1 kb flanking regions and most likely tag candidate casual genes (Supplementary Table S1).

The statistical significance of the difference in phenotype means was checked with a Student’s *t*-test between samples that were reference homozygous, alternative homozygous, and heterozygous for the identified SNPs. The difference for the TSW phenotype was significant (*p*-value <0.05) for 8 out of 49 SNPs within genes and 1 kb flanking regions and for 3 more SNPs among the rest of the identified 50 SNPs (Supplementary Table S3).

For the TSW trait, the identified SNPs were found in known genes involved in the biosynthesis and transport of nutrients and adaptation to unfavorable environmental conditions. Two SNPs (Chr4:38305135; Chr4:38305142) were within the *Ca_13125* gene encoding the diacylglycerol O-acyltransferase 1C-like protein DGAT1. DGAT is considered to be a key enzyme in the conversion of diacylglycerol (DAG) to TAG. Triacylglycerols (TAGs) are the major seed storage lipids, providing carbon and energy reserves to support seedling growth during germination [54]. TAGs are involved in mediating pollen development and sexual reproduction in many plant species (DGAT1) [52]. A study conducted in rapeseed plants (*Brassica napus* L.) showed that inhibition of DGAT1 resulted in reduced seed oil content and germination rates, as well as severe developmental abnormalities [55].

The SNP Chr1:1457542 was found within Ca_00178, which encodes the oligopeptide transporter 4 known as OPT4. In *Arabidopsis thaliana* plants, OPT4 transporter proteins are responsible for loading peptides into the plant vasculature [56].

Three SNPs (Chr2:35757170; Chr2:35759932; and Chr2:35756260) were found in the *Ca_09749* gene, encoding the NF-kappa-B-activating protein Nkap. MSA2, an orthologue of human Nkap in *Arabidopsis thaliana*, is known to be involved in the regulation of rRNA synthesis [57].

The *Ca_08059* gene contained three SNPs (Chr1:8130179; Chr1:8131436; and Chr1:8131450), which encodes a protein from the subtilase family–subtilisin-like protease Glyma18g48580. Studies on soybean *Glycine max* L. showed the role of this protein in its interaction with the GmSubPep peptide and subsequent activation of defenses in response to the presence of pathogens [58].

Our study identified an SNP known as Chr6:10613546 located in the *Ca_08536* gene encoding the E3 ubiquitin-protein ligase SINAT2 protein. In *Hordeum vulgare* L. SINAT2 was identified as a candidate gene involved in salt response [59]. Salinity limits chickpea growth and productivity by interfering with various physiological and metabolic processes [60]. On average, salinity can reduce chickpea plant growth rates by 20%, plant heights by 15%, and shoot biomass levels by 28%. It also causes unfilled pods and reduces the seed number and yield by 16% and 32%, respectively [61].

The SNPs Chr1:2511223 and Chr1:2511238 are located in the *Ca_00314* gene, encoding the acetolactate synthase 3 AHAS3. It is known that a point mutation in the chickpea AHAS1 gene results in an amino acid change from Ala205 to Val205, which makes chickpeas resistant to the herbicide imidazolinone [62]. This gene was found in the study [63] to be associated with the seeds per plant and days to maturity traits (see Suppl.tab.14 in [63]).

An SNP (Chr1:5757141) was found in the *Ca_00667* gene encoding the glutathione-dependent enzymes glyoxalase I (GLX1) gene—the activity of which plays a critical role in the detoxification of methylglyoxal [64].

## 5. Discussion

In this work, a methodology was proposed that combines AIOs to encode information on genomic features—6642 SNPs for each accession, a CNN, dictionary learning and sparse coding for feature extraction, extreme gradient boosting for phenotype prediction, and dense regression attention maps for the identification of the most import genomic factors. This approach took into account the properties of convolutional neural networks.

Two main phenotypic traits were taken as productivity indicators for modeling: the thousand-seed weight (TSW) and number of seeds per plant (SNpP). The models were trained using 10-fold cross-validation and reached an acceptable accuracy of 84% and 85% for the TSW and SNpP, respectively.

For each trait, 99 SNPs were selected as the most important for the model solution, of which 34 (for SNpP) and 49 (for TSW) were located within known genes or within 1 kb flanking regions and most likely tag candidate casual genes. The functions of some of them are known.

The difference in phenotype means was statistically significant with the Student’s *t*-test (*p*-value <0.05) employed between samples that were reference homozygous, alternative homozygous, and heterozygous for the identified SNPs for 8 out of 49 SNPs and for 23 out of 34 SNPs for the TSW and SNpP traits, respectively.

Two SNPs (Chr1:2511223; Chr1:2511238) found in this study to be important for TSW are located at the distance 85 bp and 70 bp, respectively, from the SNP (Chr1:2511308) that was found by FarmCPU to be associated with the seeds-per-plant trait in [63]. The SNP (Chr4:44945463) from this study is located 744 bp away from the SNP (Chr4:44946207) that was found by FarmCPU to be associated with plant height in [63].

The accuracy of genomic prediction achieved using the developed approach suggests the need to explore the phenotypic performance of the genotypes of interest in different environments. One of the important implications of the present research is that the application of advanced machine learning methods for the prediction of phenotypic traits from genomic data may be computationally expensive but makes it possible to find new potential candidate genes that can be further investigated for use in breeding programs.

The main limitation of the proposed approach is the need to encode the genomic data in AIOs and compute the sparse representation according to the dictionary. It should be noted that this step needs to be done once for the dataset, and the results are then used for all traits. As the proposed model predicts the phenotype from features extracted from the images, the encoding scheme that maps the genomic data to pixel intensities may influence the model performance. The three-color channel scheme utilized in this work may not be appropriate in all cases, for example, for SNPs with more allele combinations.

## 6. Conclusions

The proposed methodology efficiently encoded genomic factors as AIOs and constructed a model based on dictionary learning and sparse coding, CNN, and extreme gradient boosting that predicted the seeds number per plant and thousand-seed weight values of accessions from a chickpea dataset with an acceptable accuracy of ≈85%. In the context of chickpea breeding, our approach can help to more effectively produce varieties with desired traits; however, further studies are necessary to provide new valuable and unbiased information for assessing its methodological potential.

The most important factors that influence the model solution were identified using dense regression attention maps. The important SNPs were found in known genes or within 1 kb flanking regions and most likely tag candidate casual genes the functions of some of which that are known. The difference in phenotype means was statistically significant with the Student’s *t*-test values yielded for a fraction of these SNPs, which supports the importance of these factors for traits of interest.

Future research in the application of artificial image objects and machine learning methods regarding the identification of important SNP markers and the construction of predictive models of important agronomic traits will include the optimization of the layout of factors in AIOs. The SNPs that are not close to each other in terms of genomes may be placed to the same part of the AIO and picked up together by the convolution kernel. Our future research will also include model verification with independent datasets.

## Data Availability

The data for this publication is available at Zenodo https://doi.org/10.5281/zenodo.12755678.

## Supplementary Materials

The following are available online at https://www.mdpi.com/article/10.3390/plants13172444/s1: Table S1: SNP identified in for TSW located in gene body (GB) or 1 kb flanking regions; Table S2: SNP identified in for SNpP located in gene body (GB) or 1 kb flanking regions; Table S3: results for statistical significance of difference in phenotype means for different allele combinations.
