# Supplementary material for: Modeling Chickpea Productivity with Artificial Image Objects and Convolutional Neural Network
Source: Plants (Basel). 2024 Sep 1;13(17):2444. doi: 10.3390/plants13172444 (PMC11397516; doi:10.3390/plants13172444)
Supplement: Supplementary file 1 [file plants-13-02444-s001.zip › Supplementary table 1 for TSW.docx]

Supplementary table 1

SNPs located in gene body (GB) or 1kb flanking regions

| # | chrs | pos | Closest gene | Position relative to closest gene | Closest gene description |
| --- | --- | --- | --- | --- | --- |
| 1 | Ca4 | 25610715 | Ca_16587 | upstream 818 | uncharacterized protein |
| 2 | Ca1 | 1443614 | Ca_00176 | downstream 815 | probable cinnamyl alcohol dehydrogenase 1 |
| 3 | Ca1 | 1457542 | Ca_00178 | GB | oligopeptide transporter 4-like |
| 4 | Ca1 | 5757141 | Ca_00667 | GB | lactoylglutathione lyase GLX1-like |
| 5 | Ca1 | 5758008 | Ca_00667 | downstream 256 | lactoylglutathione lyase GLX1-like |
| 6 | Ca7 | 36708736 | Ca_13756 | GB | uncharacterized protein |
| 7 | Ca7 | 36747540 | Ca_13761 | GB | - |
| 8 | Ca4 | 4396253 | Ca_03821 | GB | kelch repeat-containing protein At3g27220-like |
| 9 | Ca4 | 4407288 | Ca_03820 | GB | uncharacterized protein |
| 10 | Ca4 | 4419313 | Ca_03818 | upstream 19 | uncharacterized protein |
| 11 | Ca4 | 38305135 | Ca_13125 | GB | diacylglycerol O-acyltransferase 1C-like |
| 12 | Ca4 | 38305142 | Ca_13125 | GB | diacylglycerol O-acyltransferase 1C-like |
| 13 | Ca4 | 38306386 | Ca_13124 | upstream 182 | casein kinase 1-like protein 1 |
| 14 | Ca1 | 1532918 | Ca_00186 | upstream 291 | uncharacterized protein |
| 15 | Ca1 | 1533118 | Ca_00186 | upstream 91 | uncharacterized protein |
| 16 | Ca1 | 1534227 | Ca_00186 | GB | uncharacterized protein |
| 17 | Ca2 | 35756260 | Ca_09749 | upstream 154 | NF-kappa-B-activating protein |
| 18 | Ca2 | 35757170 | Ca_09749 | GB | NF-kappa-B-activating protein |
| 19 | Ca2 | 35759932 | Ca_09749 | GB | NF-kappa-B-activating protein |
| 20 | Ca7 | 35344411 | Ca_17649 | GB | uncharacterized protein |
| 21 | Ca7 | 35344443 | Ca_17649 | GB | uncharacterized protein |
| 22 | Ca2 | 35410355 | Ca_09700 | upstream 548 | myosin-binding protein 3 |
| 23 | Ca2 | 35433799 | Ca_09703 | GB | P-loop nucleoside triphosphate hydrolase superfamily protein |
| 24 | Ca3 | 31520967 | Ca_12251 | GB | uncharacterized protein |
| 25 | Ca3 | 31530104 | Ca_12252 | GB | - |
| 26 | Ca3 | 31531883 | Ca_12252 | GB | - |
| 27 | Ca1 | 2510183 | Ca_00314 | upstream 11 | acetolactate synthase 3 |
| 28 | Ca1 | 2511223 | Ca_00314 | GB | acetolactate synthase 3 |
| 29 | Ca1 | 2511238 | Ca_00314 | GB | acetolactate synthase 3 |
| 30 | Ca4 | 44945463 | Ca_09157 | GB | - |
| 31 | Ca4 | 44947721 | Ca_09157 | GB | - |
| 32 | Ca4 | 17279574 | Ca_05394 | upstream 800 | - |
| 33 | Ca4 | 17303877 | Ca_05391 | GB | - |
| 34 | Ca4 | 17309656 | Ca_05391 | GB | - |
| 35 | Ca3 | 36956205 | Ca_01022 | GB | uncharacterized protein |
| 36 | Ca3 | 36970281 | Ca_01025 | upstream 579 | plasma membrane ATPase 4 |
| 37 | Ca4 | 45109176 | Ca_09171 | upstream 251 | uncharacterized protein |
| 38 | Ca4 | 45109519 | Ca_09171 | GB | uncharacterized protein |
| 39 | Ca4 | 45111471 | Ca_09171 | GB | uncharacterized protein |
| 40 | Ca2 | 31213018 | Ca_12442 | upstream 212 | WEB family protein At5g55860-like |
| 41 | Ca2 | 31235335 | Ca_12440 | GB | cellulose synthase-like protein E1 |
| 42 | Ca2 | 31235353 | Ca_12440 | GB | cellulose synthase-like protein E1 |
| 43 | Ca2 | 11431990 | Ca_18791 | GB | pyruvate kinase |
| 44 | Ca1 | 8130179 | Ca_08059 | GB | subtilisin-like protease Glyma18g48580 |
| 45 | Ca1 | 8131436 | Ca_08059 | GB | subtilisin-like protease Glyma18g48580 |
| 46 | Ca1 | 8131450 | Ca_08059 | GB | subtilisin-like protease Glyma18g48580 |
| 47 | Ca6 | 10613546 | Ca_08536 | GB | E3 ubiquitin-protein ligase SINAT2 |
| 48 | Ca6 | 10627082 | Ca_08535 | GB | gamma-tubulin complex component 3 |
| 49 | Ca6 | 10659481 | Ca_08531 | GB | protein trichome birefringence |
