# Supplementary material for: Modeling Chickpea Productivity with Artificial Image Objects and Convolutional Neural Network
Source: Plants (Basel). 2024 Sep 1;13(17):2444. doi: 10.3390/plants13172444 (PMC11397516; doi:10.3390/plants13172444)
Supplement: Supplementary file 1 [file plants-13-02444-s001.zip › Supplementary table 2 for SNpP.docx]

Supplementary table 2

SNPs located in gene body (GB) or 1kb flanking regions

| # | chrs | pos | Closest gene | Position relative to closest gene | Closest gene description |
| --- | --- | --- | --- | --- | --- |
| 1 | Ca1 | 22112036 | Ca_20639 | GB | uncharacterized protein |
| 2 | Ca1 | 25737022 | Ca_18581 | GB | 18.5 kDa class I heat shock protein-like |
| 3 | Ca1 | 25866735 | Ca_18586 | GB | PREDICTED: uncharacterized protein |
| 4 | Ca1 | 6346124 | Ca_07895 | 314 downstream | uncharacterized protein At3g61260-like |
| 5 | Ca1 | 30186094 | Ca_22469 | GB | uncharacterized protein |
| 6 | Ca1 | 7478974 | Ca_08000 | GB | biotin carboxyl carrier protein of acetyl-CoA carboxylase, chloroplastic-like |
| 7 | Ca1 | 7489046 | Ca_08002 | GB | hypothetical protein TSUD_158340 |
| 8 | Ca1 | 7497743 | Ca_08003 | 306 upstream | ER membrane protein complex subunit 10 |
| 9 | Ca1 | 26950927 | Ca_20294 | GB | pentatricopeptide repeat-containing protein At1g63330-like |
| 10 | Ca1 | 26966675 | Ca_20295 | 925 downstream | protein SEH1 |
| 11 | Ca1 | 6553315 | Ca_07920 | GB | uncharacterized protein |
| 12 | Ca1 | 19800082 | Ca_08856 | 653 upstream | uncharacterized protein |
| 13 | Ca1 | 19804162 | Ca_08855 | GB | serine/threonine-protein kinase PCRK1-like |
| 14 | Ca1 | 6354038 | Ca_07897 | GB | DEAD-box ATP-dependent RNA helicase 8 |
| 15 | Ca1 | 6354059 |  | GB |  |
| 16 | Ca1 | 6354082 |  | GB |  |
| 17 | Ca1 | 26891914 | Ca_20291 | GB | zinc-finger homeodomain protein 2-like |
| 18 | Ca1 | 7560046 | Ca_08010 | 621 upstream | Retrovirus-related Pol polyprotein from transposon TNT 1-94, partial |
| 19 | Ca1 | 7565347 | Ca_08011 | GB | lysine--tRNA ligase-like |
| 20 | Ca1 | 7592329 | Ca_08013 | 213 downstream | AP2-like ethylene-responsive transcription factor AIL1 |
| 21 | Ca1 | 31369599 | Ca_21855 | GB | protein NRT1/ PTR FAMILY 2.11-like |
| 22 | Ca4 | 3171636 | Ca_12117 | GB | internal alternative NAD(P)H-ubiquinone oxidoreductase A2, mitochondrial-like |
| 23 | Ca1 | 28473203 | Ca_18506 | GB | armadillo repeat-containing protein 7-like |
| 24 | Ca3 | 31314787 | Ca_12230 | 58 downstream | uncharacterized protein |
| 25 | Ca3 | 31315015 |  | 286 downstream |  |
| 26 | Ca3 | 31319589 | Ca_12231 | GB | uncharacterized protein |
| 27 | Ca1 | 8130166 | Ca_08059 | GB | subtilisin-like protease Glyma18g48580 |
| 28 | Ca1 | 8130179 |  | GB |  |
| 29 | Ca1 | 8131436 |  | GB |  |
| 30 | Ca1 | 34314586 | Ca_25478 | GB | LEAF RUST 10 DISEASE-RESISTANCE |
| 31 | Ca1 | 6453725 | Ca_07909 | 190 downstream | uncharacterized protein |
| 32 | Ca1 | 27024341 | Ca_20299 | GB | protein FAR1-RELATED SEQUENCE 5-like |
| 33 | Ca1 | 6155945 | Ca_00700 | 471 downstream | E3 ubiquitin-protein ligase RNF123 |
| 34 | Ca1 | 6162693 | Ca_00701 | GB | 1-aminocyclopropane-1-carboxylate oxidase |
